# Pregnancy and helminth infections

**DOI:** 10.1111/pim.12101

**Published:** 2014-09-05

**Authors:** H Mpairwe, R Tweyongyere, A Elliott

**Affiliations:** 1 MRC/UVRI Uganda Research Unit on AIDSEntebbe, Uganda; 2 Makerere University College of Veterinary Medicine, Animal Resources and BiosecurityKampala, Uganda; 3 London School of Hygiene & Tropical MedicineLondon, UK

**Keywords:** allergy, anaemia, birthweight, helminth, offspring, pregnancy

## Abstract

It has been proposed that helminth infection may be particularly detrimental during pregnancy, through adverse effects on maternal anaemia and on birth outcomes, and that anthelminthic treatment during pregnancy will therefore be particularly beneficial. However, the few treatment trials that have been conducted have given, but little support to this notion and further trials in settings of nutritional stress are needed. It has also been proposed that prenatal exposure to helminth infection has an important effect on the development of the foetal immune response. There is evidence that this may impact, long-term, upon responses to helminth and nonhelminth antigens, and to allergens. Exposure to helminths *in utero* may also have nonspecific effects that may modify the offspring's susceptibility to diseases mediated by inflammation, including metabolic disorders. The mechanisms of such effects are not known, but they deserve to be explored as current epidemiological findings suggest the possibility of primary prevention for inflammatory conditions such as allergy, through intervention during pregnancy.

## INTRODUCTION

Helminth infections are highly prevalent. In 2006, it was estimated that over a billion people were infected with one or more species of soil-transmitted helminth 1, while 200 million were infected with schistosomes 2. The prevalence of hookworm tends to increase with age 3 and, although the intensity of schistosomiasis declines with age, the prevalence remains high among young adults in endemic communities 4. Thus, in endemic settings, there is a high burden of helminth infection among pregnant women, a group already at increased risk from malaria, and susceptible to anaemia and to nutritional stress 5–8. Implications for the mother and foetus include possible physiological effects on haemoglobin levels and on foetal growth and development, and possible immunological effects on both mother and foetus. This review will examine current evidence for such effects, focusing mainly on the latter, and discussing possible mechanisms for some of the immunological effects that have been observed.

Observational studies on the effects of helminth infections in humans are fraught with strong potential for both measured and unmeasured confounding, as helminths occur in the most disadvantaged communities alongside poverty, lack of education and malnutrition. Helminth-infected pregnant women are likely to be younger, less educated and of lower socio-economic status than uninfected women 5 – all factors that may impact upon nutrition and upon health-seeking behaviour (importantly, uptake of iron and folate supplements, preventive treatment for malaria and use of bed nets) for themselves and their infants. They may come from different ethnic backgrounds and live in different environments, compared with uninfected women 5, and these factors may influence customs or behaviour, and exposure of themselves and their infants to helminths and other infections. Co-exposure of the foetus or infant to malaria may be more common for environmental reasons, or because of immunomodulating effects of helminths 9,10. Randomized clinical trials of anthelminthic treatment should help to address this and to identify reversible effects directly caused by helminths.

## ANTHELMINTHIC TREATMENT DURING PREGNANCY

Historically, pregnant and breastfeeding women have often been excluded from the treatment for helminths, due to concern about possible adverse effects of the drugs on the developing foetus. However, in 1994, the World Health Organization (WHO) recommended the treatment of pregnant women for hookworm, because of concern about hookworm-induced anaemia 11. Further, in 2002, a WHO informal consultation recommended the use of praziquantel during pregnancy and lactation, and that in schistosomiasis-endemic settings, all girls and women of child-bearing age should be included in praziquantel mass drug administration programmes 12. It was hoped that implementation of these recommendations would result in benefits including reduced maternal anaemia, increased birthweight and reduced perinatal and infant mortality. Neither of these recommendations was based on evidence of benefit or safety from controlled trials, and in the case of praziquantel, a subsequent WHO Scientific Working Group, held in 2006, called for such trials to be conducted 13. However, only a very small number of anthelminthic trials have yet been conducted among pregnant women 14–22 (Table 1), and taken together, the results are, as yet, inconclusive.

**Table 1:** Clinical trials of anthelminthic treatment during pregnancy

Country; trial name	Participants	Helminth prevalence		Intervention	Outcomes assessed	Main results	References
Entebbe, Uganda; Entebbe mother and baby study	2507	Hookworm	45%	Alb 400 mg vs. placebo pzq 40 mg/kg vs. placebo each single dose	Maternal anaemia	No effects	19–22
		*Mansonella perstans*	21%		Maternal HIV load	Reduced by alb	
		*Schistosoma mansoni*	18%		MTCT of HIV	No effects	
		*Schistosoma stercoralis*	12%		Congenital abnormalities	No effects	
		*Trichuris trichiura*	9%		Adverse birth outcomes^a^	No effects	
		*Ascaris lumbricoides*	2%		Birthweight	No effects	
					Infant mortality	No effects	
					Infant response to vaccines	No effects	
					Infant malaria, diarrhoea, pneumonia	No effects	
					Infantile eczema	Increased by alb, pzq	
					Infant cognitive development	No effects	
Freetown and Port Loko, Sierra Leone	184	Hookworm^b^	66%	Alb 400 mg vs. calcium and vitamin D single dose	Change in anaemia btw 1st & 3rd trimester	Benefit	18
		*Trichuris trichiuria*	74%		Change in iron status btw 1st & 3rd trimester	No effect	
		*Ascaris lumbricoides*	20%		Miscarriages	No effect	
					Congenital abnormalities	No effect	
Iquitos, Peru	1042	Hookworm	46%	Meb 500 mg single dose	Maternal anaemia	No effect	15
		*Trichuris trichiuria*	82%		Birthweight	No effect	
		*Ascaris lumbricoides*	64%		Low birthweight	Reduced	
					Congenital abnormalities	No effect	
					Adverse birth outcomes^a^	No effect	
Masindi, Uganda	591 STH-infected 241 comparison^c^	Hookworm	67%	Alb400 mg vs. ivc (dose according to height) vs. ivc plus alb	Helminth “cure”	Alb superior for hkwm	16
		*Trichuris trichiuria*	5%		Miscarriage, prematurity	No effect	
		*Ascaris lumbricoides*	0·5%		Congenital abnormalities	No effect	
		*Schistosoma mansoni*	4%		Maternal haemoglobin	No effect	
					Birthweight	No effect	
					Infant haemoglobin	No effect	
					Infant mortality	No effect	
Rufiji, Tanzania	3080 cluster randomized by antenatal clinic			Albendazole	Maternal anaemia (<10·5 g/dL) at term	No effect	17
					Maternal haemoglobin (Hb) at term	No effect	
					Maternal anaemia 4 months post-partum	Benefit	
					Maternal Hb 4 months post-partum	Benefit	
Lamberene, Gabon	65	*Schistosoma haematobium*	100%	Mefloquine 15 mg/kg vs. SP (3 tablets of 500/25 mg) twice during pregnancy	Helminth “cure”	Mefloquine superior	14

a Miscarriages, still births, neonatal deaths and (in Gyorkos 2006) prematurity

b Necator americanus

c only infected women were included in the treatment trial; alb, albendazole; pzq, praziquantel; meb, mebendazole; ivc, ivermectin; STH, soil-transmitted helminths; btw, between primary outcomes highlighted.

## PHYSIOLOGICAL EFFECTS OF HELMINTHS IN PREGNANCY

There is substantial evidence from studies in human subjects that certain species of helminth infection during pregnancy are associated with maternal anaemia, as reviewed by Brooker and colleagues in 2008 23, and observed in several subsequent studies 24–27. It is likely that heavy intensities of hookworm and *Trichuris trichiura* are particularly important 28. However, only two of five trials assessing this outcome have shown a benefit of treatment for soil-transmitted helminths during pregnancy (Table 1) 17,18. In a trial setting, it is almost imperative to provide adequate iron and folate supplementation, and (where relevant) presumptive treatment for malaria during pregnancy, as standard of care during pregnancy. This may override some of the potential benefits of anthelminthics: Torlesse and colleagues found greater benefit of haematinic supplementation than of albendazole in a hookworm-endemic region of Sierra Leone 18.

Studies in mice suggest that maternal worm infections influence foetal linear growth and the growth of lymphoid tissues and bone 29,30. There are also observational studies in human subjects suggesting that certain maternal helminth infections adversely affect birthweight and perinatal and infant mortality and that anthelminthics during pregnancy may be beneficial for these outcomes 31–35 [reviewed by Freidman 35 and Imhoff-Kunsch 36], but trials to date have failed to confirm that these associations are causal by showing a benefit of treatment (Table 1). Anaemia, itself, is an important cause of adverse birth outcomes 37–39, and if effects of helminths on anaemia are obscured by supplementation with haematinics this may also obscure possible benefits of anthelminthics mediated by reductions in anaemia 34.

If the hypothesis that anthelminthic treatment during pregnancy can have important benefits for maternal and infant birth outcomes is to be rigorously tested, and the policy of routine anthelminthic treatment during pregnancy is to be validated, large trials are still needed in a range of settings. It may be that anthelminthics will be found most useful in settings in which anaemia is prevalent, and where there is nutritional stress and limited access to haematinic supplementation. In such settings, single dose benzimidazoles, [which are easy to administer and have been highly effective against hookworm in most trials to date 15,16,18,19] may have benefits for birth outcomes. No trials have yet demonstrated benefits of praziquantel treatment during pregnancy for maternal anaemia or for birth outcomes, but a further placebo-controlled trial of praziquantel treatment for *Schistosoma japonicum* during pregnancy is in progress and scheduled for completion this year 40. This will be of particular interest because *S. japonicum* has been shown to be associated with inflammation in maternal, placental and foetal tissues 41, and studies *in vitro* suggest that exposure of trophoblast cells to *S. japonicum* egg antigen may result in impaired placentation 42.

## EFFECTS OF PREGNANCY ON SUSCEPTIBILITY TO HELMINTHS AND ON THE RESPONSE TO THEIR TREATMENT

Pregnancy results in major hormonal and nutritional changes, and in immunological changes allowing successful allograft retention 43–45. In mammals other than humans, there is a well-recognized increase in susceptibility to helminths, or at least to helminth egg production, around parturition. Immune modulation resulting from hormonal changes (such as a reduction in circulating cortisol) 46, or nutritional changes, such as protein deficiency 47 may contribute to this effect. In humans, data are conflicting, with studies showing increased 7 or similar 48 helminth egg counts between pregnant and nonpregnant women.

Studies in mice also suggest that pregnancy is associated with increased schistosome-induced pathology, related to reduced parasite-specific interferon (IFN)-γ but increased interleukin (IL)-4 production 49. If similar effects were demonstrated in humans, this would be a further strong argument for emphasizing schistosomiasis treatment in young, and pregnant, women. In the Entebbe Mother and Baby Study (EMaBS), a trial of albendazole and praziquantel among pregnant women in Uganda 50, we examined effects of pregnancy on the immune response to schistosomiasis by comparing responses during pregnancy and after delivery in placebo recipients. Interleukin-2, IL-4 and IL-10 responses to schistosome worm and egg antigens were similar during pregnancy and after delivery, whereas IFN-γ, IL-5 and IL-13 responses increased after delivery, suggesting that pregnancy in humans is indeed associated with a modified profile of antischistosome cellular responses 51. Antischistosome antibody levels were also lower during pregnancy 52. Further studies to investigate whether these immunological differences are associated with differences in the progression of pathology would be justified.

Treatment of schistosomiasis with praziquantel in nonpregnant human subjects is associated with release of worm antigen into the blood stream and with a boost in cellular and antibody responses to worm and egg antigen 53. We observed a similar, but smaller boost in responses following treatment during pregnancy, when compared to treatment after delivery 51,52. This may be important for several reasons. First, the immune response is thought to synergize with the direct effects of praziquantel, helping to kill the parasites 54. A reduced boost might mean that treatment in pregnancy was less effective. However, we found no difference in egg reduction following treatment during pregnancy compared with treatment after delivery in our study 51. Second, the sudden immunological changes following treatment might have implications for the success of the pregnancy. Successful pregnancies are associated with a placental microenvironment that is dominated by a type two cytokine milieu, characterized by increased production of IL-4 and IL-10, and decreased production of IFNγ and IL-2 55. Animal models of pregnancy have shown that increased systemic and placental IFNγ and TNFα levels are associated with poor pregnancy outcomes 56,57. Reassuringly, in EMaBS, praziquantel treatment of *Schistosoma mansoni* during pregnancy was not associated with an increased rate of miscarriage or still birth 19. However, the number of women with schistosomiasis in the study was fairly small (458), so its power to detect differences in these rates was limited. Vigilance regarding birth outcomes will be important as the 2002 WHO recommendations 12, to use praziquantel during pregnancy and lactation, are more widely implemented.

## IMMUNOLOGICAL EFFECTS OF HELMINTHS ON PREGNANT WOMEN

Helminth infections modulate the host immune response through a variety of mechanisms 58. These allow long-term survival of the parasite within the host, while minimizing damage to host tissues. They also have implications that may be important during pregnancy.

First, they may impact upon susceptibility to heterologous infections. Effects on susceptibility to malaria might be especially relevant during pregnancy, given its adverse impact on pregnancy outcomes 59. Positive associations between hookworm and malaria have been reported 9,60,61, while *S. haematobium* and *Ascaris lumbricoides* have been associated with lower malaria parasite densities 60 and lower malaria risk 61, respectively. While these associations might be confounded by environmental and behavioural factors, measured confounders failed to explain the positive hookworm malaria and *Mansonella* malaria associations observed in our study 9, and immunological mechanisms are also possible.

Second, they may impact upon maternal metabolism – for example glucose metabolism 62. A recent study in mice showed that classical activation of macrophages in adipose tissue (favouring inflammatory immune responses) was associated with glucose intolerance, while alternative activation was associated with enhanced glucose tolerance. Eosinophils in adipose tissue were shown to be a key source of IL-4 for the induction of alternatively activated macrophages, and eosinophils induced by helminth infection were shown to produce a sustained improvement in glucose tolerance 62. Similar effects in humans might be beneficial in relation to gestational diabetes.

## IMMUNOLOGICAL EFFECTS OF HELMINTHS DURING PREGNANCY ON THE FOETUS

Arguably, the most important potential impact of worm infections during pregnancy is upon long-term outcomes in the offspring.

### Effects on helminth-specific responses

There is considerable evidence from studies in both animal models 63–65 and humans 65–70 that in utero exposure to helminths can influence susceptibility to the same helminth species later in life. For example, in 1991, Lammie and colleagues, working in an area endemic for bancroftian filariasis, showed that maternal, but not paternal, filarial infection was associated with childhood infection and that children of infected mothers had reduced cellular responses to microfilariae compared with children of uninfected mothers 66. Other studies supported this result 67. Subsequently, it was shown that children of infected mothers who were sensitized *in utero* to filarial antigen showed similar immunological and clinical outcomes to unexposed infants, but that children who were exposed *in utero* and not sensitized (suggesting the acquisition of tolerance) were slower to develop detectable cellular responses and more likely to become microfilaraemic than either sensitized or unexposed infants 71,72. These results suggest an immunological explanation for the epidemiological observations. There is evidence for similar effects of intrauterine exposure in humans for onchocerciasis 69, schistosomiasis 70 and soil-transmitted helminth infection 68. While increasing susceptibility, the tolerogenic effects of prenatal exposure may reduce inflammation-induced pathology, resulting in an improved outcome for both helminth and host.

### Effects on responses to heterologous vaccines and infections

Observational studies have also suggested that exposure to worm infections during pregnancy can alter the offspring's response to unrelated antigens, including vaccines, infectious diseases and allergens. A study in Kenya found that infants exposed and sensitized to schistosomiasis and filarial worms in utero showed a Th2 bias in their immune responses to BCG vaccine, while infants who were exposed but not sensitized showed a Th1 profile of response 73. Such an effect might contribute to explaining the poor efficacy of BCG immunization in tropical developing countries – one of the settings in which protection against tuberculosis is most needed 74,75. In the EMaBS cohort, in which we compared responses following BCG and tetanus immunization in infants of helminth-infected and helminth-uninfected women, we did not observe effects on the infant Th1 or Th2 response to mycobacterial antigens, but treatment with albendazole reduced Th2 responses to tetanus toxoid among the infants of mothers with hookworm 22. Why was this effect seen for the response to tetanus immunization, but not for the response to BCG? One possibility is that concurrent prenatal exposure to helminths and to the heterologous antigen of interest may be important for the helminth effects. EMaBS women received tetanus immunization during pregnancy, providing a potential opportunity for exposure of the foetus to this antigen 76; indeed the infant cytokine response to tetanus immunization increased with the number of doses that the mother received during pregnancy, suggesting that prenatal priming of the infant antitetanus response had occurred 77. We also found that infants of mothers with *M. perstans* infection had elevated IL-10 responses to both BCG-related and tetanus antigens, but these associations were seen only among infants of mothers who received no albendazole, suggesting that prenatal exposure to albendazole, or to its effects on maternal infections, blocked the ability of maternal *M. perstans* to promote regulatory responses to heterologous antigens in the infants 77.

Besides tuberculosis, HIV infection and malaria are among the most important co-infections in helminth-endemic settings, and there is considerable controversy as to the effects of helminths on susceptibility to both 78. Prenatal exposure to maternal helminths has been postulated to increase the risk of vertical HIV transmission by activation of foetal lymphocytes, increasing their susceptibility to HIV infection. A small observational study in Kenya (among 44 HIV-exposed infants, 13 of whom were HIV infected) supported this hypothesis with an odds ratio, adjusted for maternal malaria [aOR (95% confidence interval)] of 7·3 (2·4–33·7) for any maternal helminth vs. none 79. In fact, the association was prominent among infants of mothers with filariasis, but not for other helminth species. In EMaBS, we studied the association between vertical HIV transmission and maternal helminth infection among 203 HIV-exposed babies (34 HIV-infected), and there was no statistically significant association with helminth infection (aOR for all helminths combined, adjusted for the use of nevirapine for prevention of mother-to-child HIV transmission and for vaginal delivery vs. Caesarian section, 2·23 (0·86–5·79); K. Jacquline, M.E. Alison, unpublished data). However, anthelminthic treatment during pregnancy showed no benefit for this outcome 22; thus, no firm conclusion can yet be reached as to the effects of maternal helminths on vertical HIV transmission.

In EMaBS, we also investigated associations between maternal helminth and maternal malaria infection and infant susceptibility to malaria. We found that maternal worm infections, including hookworm, *M. perstans* and *S. mansoni* were associated with increased risk of malaria incidence in the offspring 10. These associations persisted after adjusting for potential confounders (maternal age, education, parity, net ownership, socio-economic status, maternal malaria and human immunodeficiency virus infections, and location of residence) and after stratifying for maternal malaria infection status. In fact, there was a suggestion that the effect was stronger among infants of mothers who had malaria during pregnancy, again suggesting that concurrent exposure to maternal malaria and to maternal helminths might be important for helminth-induced effects on the offspring's response to malaria, as discussed above in relation to tetanus immunization.

### Effects on allergy-related disease outcomes

The most striking findings of EMaBS was that maternal albendazole during pregnancy was associated with an increased incidence of infantile eczema 20 and of eczema in the first 5 years of life 21. This effect was not confined to the infants of mothers with susceptible worm infections (hookworm, *Ascaris, Trichuris* and *Strongyloides*), so the mechanism of the effect of albendazole was not clear. However, maternal hookworm showed a significant inverse association with eczema in childhood, which increased with intensity 80, and maternal hookworm modified the effect of classical risk factors for childhood eczema, including maternal history of eczema and childhood atopy (H. Mpairwe, J. Ndibazza, E.L. Webb, M. Nampijja, L. Muhangi, B. Apule, S. Lule, H. Akurut, D. Kizito, M. Kakande, F.M. Jones, C.M. Fitzsimmons, M. Muwanga, L.C. Rodrigues, D.W. Dunne, A.M. Elliott, unpublished data). Maternal treatment with praziquantel was associated with an increased incidence of infantile eczema among infants whose mothers were infected with *S. mansoni* during pregnancy 20. Together, these findings strongly suggest that prenatal exposure to maternal helminth infection protects against infantile eczema and against the adverse effects of other exposures on this outcome. Whether this effect persists into later childhood, and influences risk of asthma, is currently under investigation.

## MECHANISMS BY WHICH MATERNAL HELMINTH INFECTION MIGHT INFLUENCE OUTCOMES IN THE FOETUS

**Figure 1:**
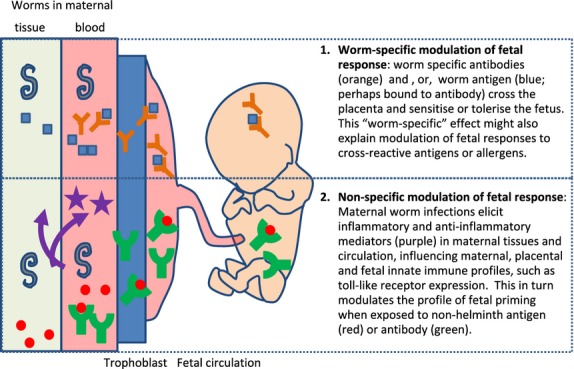
Possible mechanisms by which maternal helminth infections might influence foetal immunological development.

The mechanisms by which maternal helminth infection can influence outcomes in the foetus are not known. Investigation of these mechanisms might shed important light on the development on the infant immune response and inform the development of possible interventions for the primary prevention of immunologically mediated conditions, such as allergy 81. Some possible mechanisms are summarized in Figure 1 and discussed below.

### Foetal exposure and sensitization to foreign antigens

It is clear that the human foetus can become sensitized to parasite 73,82–88 and vaccine antigens 76 *in utero,* and at first sight, this is surprising as most macromolecules and immune complexes in the maternal circulation are broken down by macrophages in the trophoblast, providing amino acids for the growth of the placenta and foetus and preventing transfer to the foetal circulation 89. However, filarial 87 and schistosome 90–92 antigens have been detected in cord blood in humans. Similarly, there is evidence that tetanus toxoid 93 and malaria antigen 94 can cross the placenta. Studies in mice also suggest that mycobacterial antigen can cross the placenta 95, and although there are differences in placentation between mice and humans, there is evidence of prenatal sensitization of the human foetus to mycobacterial antigens *in utero* 73. How such antigens cross the placenta is not known. One possibility is that the antigens are transported bound to IgG antibody, protected by the foetal immunoglobulin G receptor (FcRn): this is supported by studies showing that the transfer of tetanus toxoid, or of malaria antigen, is dependent upon the presence or concentration of specific antibody in maternal blood 94, but the precise process of transfer is not fully understood.

With regard to helminth-specific immune responses, epidemiological evidence discussed above suggests that *in utero* sensitization results in down-regulated responses among the offspring, on encountering the homologous antigen. As reviewed by Dauby and colleagues 96, this may be due to a bias in the foetal and neonatal immune response towards the development T-regulatory and T-helper (Th)17 responses, rather than Th1 responses. The effects of helminth exposure might be analogous to findings for effects of placental malaria: *in vitro* depletion of CD4 + CD25hi regulatory T cells in cord blood demonstrated their contribution to suppression of neonatal responses to malaria antigens in malaria-exposed infants 97.

### Transfer of antigen-specific idiotype antibodies across the placenta

In addition to the possibility that antibodies facilitate the transfer of antigen across the placenta, there is evidence that maternal antibody idiotypes specific for helminth antigens can be transferred across the placenta and can themselves induce sensitization of the foetus in both animal models 64 and humans 98. In the case of schistosomiasis, studies in mice suggest that, by promoting IFN-γ responses in the neonate, *in utero* sensitization down-regulates the (Th2-mediated) granuloma formation associated with pathology 64.

### Non-helminth-specific effects

These possible mechanisms largely provide explanations for effects of maternal helminth infections on the offspring's response to the same helminth infection. How might maternal helminth infection influence the foetal response to unrelated antigens?

### Cross-reactivity between helminth immunogens and allergens

In the case of allergens, one possibility may be cross-reactivity between helminth antigens and allergens. There are many structural similarities between allergens and metazoan parasite immunogens 99, and IgE cross-reactivity has been demonstrated in several cases – for example, in human subjects, IgE cross-reactivity has been described between proteins of the nematodes *Anisakis Simplex* 100 and *A. lumbricoides* 101 and the allergen *Dermatophagoides pteronyssinus*; IgE, IgG and IgG4 cross-reactivity has been demonstrated between filarial antigen and cockroach allergen 102. IgE cross-reactivity has also been demonstrated between peanut allergen and schistosome egg antigen in a setting in Ghana where almost no clinical peanut allergy occurs and this has been shown to be attributable to cross-reactive carbohydrate determinants, rather than protein antigens 103. We hypothesize that intrauterine exposure to cross-reactive parasite immunogens might result in foetal acquisition of tolerance to allergens, or foetal sensitization to related, parasite-specific IgG idiotypes might modulate the foetal allergen-specific response to a nonpathogenic profile.

### Effects on innate immune responses

Alternatively, maternal helminth infection might influence the foetal innate immune system in such a way as to modulate the response induced on co-exposure to a wide range of heterologous antigens including malaria, tetanus and mycobacterial antigen, as well as allergens. Allergologists have explored this possibility to investigate how prenatal exposure to a barn environment might influence the offspring's risk of allergy-related disease. Using a mouse model of experimental allergic airway inflammation, Conrad and colleagues showed that low-grade inflammation in the maternal lung induced by the cowshed-derived bacterium *Acinetobacter lwoffii* was associated with reduced susceptibility to experimentally induced asthma in the offspring and that this was mediated by up-regulation of maternal Toll-like receptor (TLR) expression and circulating pro-inflammatory cytokines, and down-regulation of placental TLR expression 104,105. Helminths also have systemic effects on circulating inflammatory mediators and TLR expression and might influence placental and foetal antigen-presenting cell profiles in a related manner 106.

## CONCLUSION

Helminth infection during pregnancy is common, but the benefits of anthelminthic treatment during pregnancy are not yet clear either for the mother or for the offspring. On the other hand, there is strong epidemiological evidence that prenatal exposure to maternal helminth infection tends to down-modulate the offspring's response following infection, resulting in increased susceptibility to the same helminth species, but reduced pathology. Thus, an unwanted consequence of partially successful intervention against helminths may be increased susceptibility to helminth-induced pathology in some endemic settings as prevalence during pregnancy declines. Of more interest, however, is the increasing evidence that prenatal exposure to helminths may influence the offspring's susceptibility to other conditions, and particularly to allergy-related disease. The mechanisms of this effect deserve urgent investigation as they may indicate possible routes to the primary prevention, or management, of these conditions.
